# Macro‐ and microclimatic interactions can drive variation in species' habitat associations

**DOI:** 10.1111/gcb.13056

**Published:** 2015-11-26

**Authors:** Rachel M. Pateman, Chris D. Thomas, Scott A. L. Hayward, Jane K. Hill

**Affiliations:** ^1^ Department of BiologyUniversity of YorkYorkYO10 5DDUK; ^2^ Stockholm Environment InstituteUniversity of YorkYorkYO10 5DDUK; ^3^ School of BiosciencesThe University of BirminghamEdgbastonBirminghamB15 2TTUK

**Keywords:** climate change, invasion, Lepidoptera, niche breadth, *Pararge aegeria*, range expansion, speckled wood

## Abstract

Many species are more restricted in their habitat associations at the leading edges of their range margins, but some species have broadened their habitat associations in these regions during recent climate change. We examine the effects of multiple, interacting climatic variables on spatial and temporal patterns of species' habitat associations, using the speckled wood butterfly, *Pararge aegeria*, in Britain, as our model taxon. Our analyses reveal that this species, traditionally regarded as a woodland‐dependent insect, is less restricted to woodland in regions with warmer winters and warmer and wetter summers. In addition, over the past 40 years of climate change, the species has become less restricted to woodland in locations where temperature and summer rainfall have increased most. We show that these patterns arise mechanistically because larval growth rates are slower in open (i.e. nonwoodland) habitats associated with colder microclimates in winter and greater host plant desiccation in summer. We conclude that macro‐ and microclimatic interactions drive variation in species' habitat associations, which for our study species resulted predominantly in a widening of habitat associations under climate change. However, species vary in their climatic and nonclimatic requirements, and so complex spatial and temporal patterns of changes in habitat associations are likely to be observed in future as the climate changes.

## Introduction

The climate is changing (IPCC, 2013) and a suite of biological responses have been observed, including changes in species' phenologies (Roy & Sparks, 2000) and spatial distributions (Chen *et al*., 2011). Evolutionary responses (Bradshaw & Holzapfel, 2006) and changes in biotic interactions (Berg *et al*., 2010; Traill *et al*., 2010) have also been observed, and all of these responses may lead to changes in community composition and ecosystem functioning (Montoya & Raffaelli, 2010; Walther, 2010). One response to climate change that has received little attention, however, is changes in species' habitat associations. These associations are important as they determine the amount of habitat available to a species, which directly impacts the fraction of a landscape that can be occupied and hence the dispersal and metapopulation dynamics of species in patchy landscapes. Further, habitat availability affects rates of range expansion at species' leading edges in response to climate change (Hill *et al*., 2001; Wilson *et al*., 2009); broader habitat associations result in greater habitat availability, hence larger population sizes and smaller distances between habitat patches and so more rapid rates of range expansion (Thomas *et al*., 2001; Wilson *et al*., 2010). Habitat associations also have implications for how land management could affect the distribution of species and hence have impacts on conservation management decisions. Yet despite their importance, factors determining the habitat associations of species and how these vary over space and time are poorly understood.

Many species show spatial variation in their habitat associations in relation to geographical variation in climate (Anthes *et al*., 2008; Ashton *et al*., 2009) and often become more restricted to a narrower set of habitat types at range margins where climatic conditions are marginal for the species (Thomas *et al*., 1999; Lennon *et al*., 2002; Oliver *et al*., 2009). This variation might arise if species are restricted to the habitats which provide microclimatic conditions that allow survival in regions where macroclimatic conditions are generally unsuitable, for example particularly warm habitats at cool leading‐edge range margins (Cherrill & Brown, 1992; Thomas, 1993; Thomas *et al*., 1999), or protection from exposure to extreme high temperatures at warm trailing‐edge range margins (Suggitt *et al*., 2011; Scheffers *et al*., 2014). Variation in species' habitat associations may also arise indirectly, for example, if herbivores become restricted to host plants growing in more humid habitats in situations with low rainfall (Anthes *et al*., 2008). species' habitat associations have also been shown to vary through time (Shreeve, 1984), with a wider range of habitats utilized during climatically favourable seasons (Roy & Thomas, 2003) or years (Suggitt *et al*., 2012). Furthermore, trends in microhabitat and host plant associations have been detected over recent decades at species' cool range boundaries as conditions have warmed, for example relaxing associations with equator‐facing slopes (Thomas *et al*., 2001; Davies *et al*., 2006), and/or increasing the number of host plant species utilized (Pateman *et al*., 2012).

**Figure 1:**
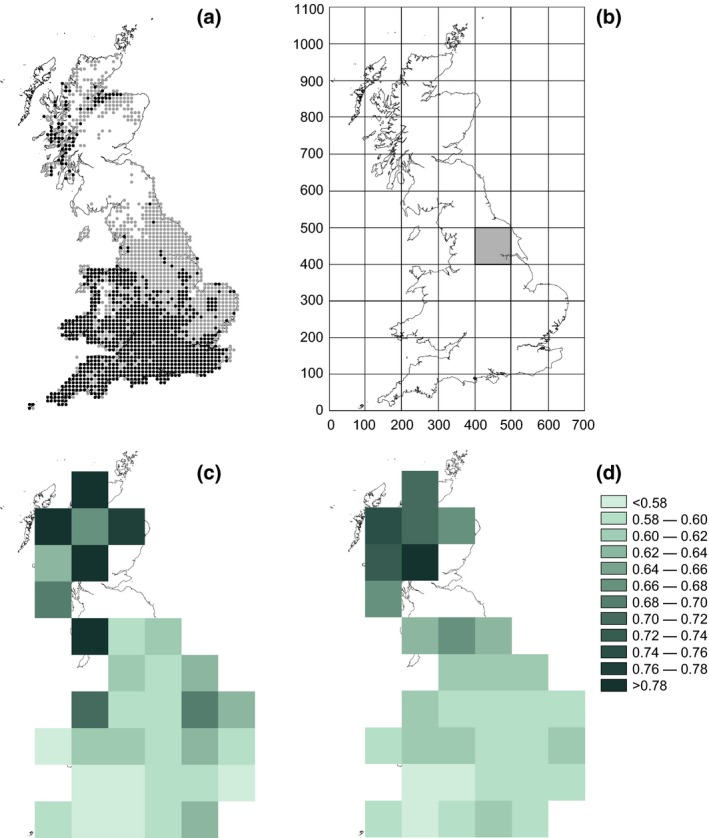
Distribution and habitat associations of *Pararge aegeria* in Britain. (a) 10 km × 10 km grid squares occupied by *P. aegeria* in 1970–1982 (Heath *et al*., 1984; black symbols) and 2005–2009 (Fox *et al*., 2011; grey symbols). (b) 100 km × 100 km UK Ordnance Survey grid squares used in the analysis of *P. aegeria*'s habitat associations. Shaded square shows location of all field experiments. (c) and (d) Strength of *P. aegeria*'s association with woodland (*W*_*i*_) in 100 km grid squares throughout Britain for period 1970–2009. A value of *W*_*i*_ = 1 indicates that all individuals were observed in woodland, a value of *W*_*i*_ = 0 means all individuals were observed outside of woodland, and a value of *W*_*i*_ = 0.5 means that individuals were equally likely to be found in woodland and nonwoodland habitats (controlling for recording effort). Observed values ranged between 0.56 and 0.81. (c) Shows observed values and (d) fitted values from the minimal adequate model (see details in text). 100 km grid squares without values are those outside of the range of *P. aegeria* (a and b) or with insufficient *P. aegeria* records to calculate a *W*_*i*_ value. Country maps created using ‘blighty’ R package (Lucy, [Link]).

Thus far, studies of climate‐driven microhabitat associations have focussed on the effects of single climatic variables (usually temperature) on species' habitat associations (e.g. Thomas *et al*., 1999; Ashton *et al*., 2009; Pateman *et al*., 2012), and multiple climate drivers have not been considered. Furthermore, habitat shifts have generally been studied within the species' favoured habitat type (e.g. shifts within grasslands from southerly‐facing slopes to other aspects), and it is unclear whether shifts to different habitat types may occur (Oliver *et al*., 2012; Suggitt *et al*., 2012). In addition, the underlying mechanisms driving patterns of local habitat associations have rarely been explained. To address these issues, we investigate the habitat associations of *Pararge aegeria* (speckled wood butterfly), which reaches its leading‐edge range margin in Britain and has expanded its distribution here in recent decades (Fig. 1a). This species generally favours woodland but is also known to use more open nonwoodland habitats (Asher *et al*., 2001; Merckx *et al*., 2003), and hence provides an opportunity to examine the role of climate in driving shifts between different habitat types. Furthermore, there is some evidence that *P. aegeria* is more restricted to its favoured habitat (woodland and other shady locations) in both the coolest (Hill *et al*., 1999; Asher *et al*., 2001; Gibbs *et al*., 2011) and hottest (Suggitt *et al*., 2012) parts of its range, implying that multiple climate factors affect its habitat associations.

We analyse the extensive historical distribution data that are available for this species in Britain, combined with new field and laboratory experiments, to examine the mechanisms driving geographical and temporal patterns of habitat associations. We test the hypotheses that (1) our study species becomes more restricted to closed habitats in locations with colder winters and drier summers because these habitats buffer against extreme temperatures and drought (Chen *et al*., 1993; Suggitt *et al*., 2011); (2) changes in habitat associations through time mirror variation in space, that is in locations where winters have become warmer and summers wetter, the butterfly has become less restricted to closed habitats; and (3) variation in habitat associations reflect the effects of winter cold and summer drought on individual survival and performance, with lower survival and poorer performance in open (grassland) habitats compared with closed (woodland) habitats.

## Materials and methods

### Spatial and temporal variation in species' habitat associations

#### Quantifying the association of *P. aegeria* with its favoured (woodland) habitat

We analysed butterfly distribution data from Butterfly Conservation and the NERC Centre for Ecology & Hydrology (see Acknowledgements) from 1970 to 2009 to test whether the association of *P. aegeria* with its favoured woodland habitat varies in space and time. For spatial analyses, we computed an ‘index of association’ of the butterfly with woodland for each 100 km × 100 km UK Ordnance Survey grid square (hereafter termed ‘100 km grid square’) in Britain (Fig. 1b; *n* = 52 100 km grid squares with some land cover; total land area varies as some coastal squares also include sea). We assigned fine‐scale 100 m × 100 m occurrence records (hereafter termed ‘100 m records’) of *P. aegeria* for the period 1970–2009 as being ‘woodland’ or ‘nonwoodland’ records using 25 m × 25 m resolution land cover data (Land Cover Map 2000; Fuller *et al*., 2002). We classified 100 m records as ‘woodland’ records if there was any woodland (defined in the Land Cover Map as deciduous, coniferous and mixed woodland, open birch, scrub, felled plantations and new plantations) within the 100 m × 100 m grid square of the record. We calculated the proportion of 100 m *P. aegeria* records that were woodland records within each 100 km grid square. To control for potential variation in recorder effort between habitats, we used records of other butterfly species as evidence that locations had been surveyed. All 100 m records of any butterfly species were assigned as being either woodland or nonwoodland records using the same methodology as for *P. aegeria* records. These fractions were used to compute the proportion of *P. aegeria* records in woodland (*W*), after accounting for differences in the number of recorded squares in woodland and nonwoodland habitats, following Eqn. (1). The fractions were calculated using only ‘unique’ records; that is, if *P. aegeria* had been recorded in the same 100 m × 100 m square multiple times throughout the period 1970–2009, the square was only counted once (*n* = 102 972 unique 100 m *P. aegeria* records) and if multiple butterfly species were observed in a 100 m × 100 m square, the square was only counted once (*n* = 377 442 100 m survey locations). Thus, *W* in Eqn. (1) represents the expected proportion of *P. aegeria* records that were in woodland if woodland and nonwoodland had been equally well recorded (with *W *=* *1 if all *P. aegeria* individuals were in woodland, and *W *=* *0 if all individuals were in nonwoodland).

(1) $$W=\frac{\frac{n}{a}}{\frac{n}{a}+\frac{m}{b}}$$

*W*, proportional occurrence of *P. aegeria* in woodland; *n*, total number of *P. aegeria* woodland records in a 100 km grid square; *m*, total number of *P. aegeria* nonwoodland records in a 100 km grid square; *a*, total number of woodland recorded squares for any butterfly species in a 100 km grid square; *b*, total number of nonwoodland recorded squares for any butterfly species in a 100 km grid square.

We computed a *W*_*i*_ value for each 100 km grid square, *i*. For temporal analyses, an index of association of *P. aegeria* with woodland was calculated using the same method, but instead of pooling data across all years, a *W*_*ij*_ value was calculated using unique records for each 100 km square *i* in each year *j* (1970–2009).

#### Climate variables

Climate variables were derived for the period 1970–2006 from monthly 5 km × 5 km grid square resolution data for mean temperature (°C) and total rainfall (mm) (UKCP09 data, MetOffice, 2009). We calculated four bioclimate variables important for butterfly survival and growth: mean winter (December to February, prior to the adult butterfly's emergence) temperature and rainfall, and mean summer (June to August), temperature and rainfall. These 5 km × 5 km data were averaged for 100 km squares across all years and for each year separately to provide estimates of climatic conditions for spatial and temporal analyses, respectively.

#### Spatial analysis

Spatial variation in the strength of *P. aegeria*'s association with woodland in relation to climate was analysed using logistic regression, with *W*_*i*_ the response variable, and winter and summer temperature and rainfall the four explanatory variables. Only 100 km grid squares with ≥20 unique 100 m records of *P. aegeria* were included in the analysis to ensure a robust calculation of association with woodland (average number of *P. aegeria* records per 100 km grid square = 2055; number of 100 km grid squares included in analysis = 34). Analyses were undertaken in r (R Development Core Team, 2007), with quasibinomial errors when necessary (Crawley, 2007). Winter and summer temperature variables are strongly correlated (Pearson's correlation coefficient = 0.76), as are rainfall variables (*r* = 0.93), and so we ran separate logistic regression models of *W*_*i*_ for each of the four climate variables, as well as a single model with all four climate variables and their interactions included. Model reduction was performed with nonsignificant terms removed in turn, beginning with the highest order interactions and working down to main effects. Main effects were retained if they were not significant but were present in a significant interaction term. For all models, we used Moran's *I* to test for spatial autocorrelation of residuals.

#### Temporal analysis

First, we ran separate logistic regressions for each 100 km grid square with *W*_*ij*_ as the response variable and year as the explanatory variable. The slope coefficients from each of these models were used to assess changes in habitat associations in each 100 km grid square over time and used as response variables in the subsequent analysis. Only 100 km grid squares with ≥20 *P. aegeria* records in ≥10 years were included in the analysis (*n* = 20 100 km grid squares). We ran linear regressions for each of the four climate variables using data from 1970 to 2006. The slope coefficients from the year‐on‐climate models examined how the climate had changed over time in each 100 km grid square and were then used as explanatory variables in the subsequent analysis. We then regressed the rate of change in climate over time in each 100 km grid square (slope coefficients from year‐on‐climate models) on rate of change in strength of association with woodland over time (slope coefficients from year‐on‐*W*_*ij*_ models). Separate models were performed for each climate variable separately as well as a model that included all those climate variables that best explained spatial variation in the study species' association with woodland.

### Experimental investigation of temperature and rainfall effects on habitat associations

#### Field experimental design

The F1 larval offspring of wild‐caught adult *P. aegeria* were reared in closed and open sites in the UK over winter 2008–2009 (1 woodland and 1 grassland site) and summer 2009 (3 woodland and 3 grassland sites) to examine survival rates and larval performance in different habitats (full details in Supporting Information). Adult *P. aegeria* butterflies were caught in a woodland site close to the northern range boundary of the species in England (Ordnance Survey grid square SE53; Fig. 1a, b) in August 2008 and July 2009 and kept separately to lay eggs on potted *Poa pratensis* host plants. Larvae were transferred to fresh potted *P. pratensis* host plants once they had reached second larval instar (due to the risk of damaging first instar larvae) and placed at sites close to where the adult females were caught (SE63, SE65, SE53; Fig. 1b). In the winter experiment, pots (40 in each site, 5–15 larvae in each pot in a split brood design; that is, larvae from one female contributed to one pot in each site) were set up in September and left overwinter until the following June, with plants replaced when they had been eaten. In the summer experiment, pots (seven in each site, five larvae in each pot with larvae from each female split evenly between treatments but assigned randomly to pots within treatments) were set up at the beginning of August and then left to desiccate in the field until the end of September. Larval development time was calculated as the time (weeks for winter experiment; days in summer) from placing larvae into the field to pupation. Fresh pupal mass and adult dry mass were measured, and larval growth rates were calculated as pupal fresh mass divided by development time. Larval survival was calculated for each pot as the percentage of larvae that pupated (summer and winter), and/or were still alive as larvae at the end of the experimental period (summer). For the other variables, average values were taken across all individuals in a pot for analysis. Due to low rates of pupation in the summer experiment, and therefore low numbers of pots with development time and pupal weight data available, we pooled data across sites and compared larval performance for all pots in woodland with all pots in grassland using *t*‐tests (with arcsine square root transformation of percentages for the survival analysis).

Four temperature data loggers were suspended at 30 cm above the ground at each site during winter and summer experiments and used to compute microclimate variables. Over winter, variables were chosen which are known to affect survival and performance in insects, corresponding to severity, duration and fluctuations in cold exposure. Over winter (including autumn and spring) and summer, growing degree days above 5 °C (GDD5) were calculated (by summing hourly temperature readings above 5 °C for each day, dividing by 24 and then summing these daily values for the experimental period) as a measure of thermal availability for larval development. *P. aegeria* larvae overwinter at the base of tufts of grass and, in some instances, under snow cover, both of which may provide some microclimatic protection (Morecroft *et al*., 1998; Groffman *et al*., 2001). Thus, while our study variables may not be directly representative of the overwintering microclimate of individuals, they do represent general differences in overwintering conditions between woodland and grassland sites. In summer, samples of grass from each pot (total *n* = 38 due to loss of some pots) as well as from the vicinity of the experiment (*n* = 28 per site) were taken to compute water content. *T*‐tests and Mann–Whitney *U*‐tests were used to compare water content of grass between woodland and grassland.

#### Laboratory experimental design

We investigated the lethal and sublethal effects of severity and duration of cold exposure on *P. aegeria* larvae in controlled conditions in the laboratory (see Supporting Information for full details). Larvae were kept in controlled cabinets at 13 °C and fed on potted *P. pratensis* host plants. Prior to experimental treatments, larvae were prevented from feeding for 3 days to minimize the amount of food in the gut (Sinclair *et al*., 2003). Groups of 10 larvae were placed in flat‐bottomed glass tubes in a tray of antifreeze (to maintain a stable temperature) and transferred into incubators where the temperature was reduced to −5 °C or −10 °C at a rate of 1 °C per minute. A total of three tubes were then removed from the −5 °C incubator after 2, 4, 6 and 8 days and from the −10 °C incubator after 1, 2, 3 and 4 days. These resulted in freezing degree days similar to the maximum experienced in the field experiment (Tables S1 and S2). While these treatments do not represent the variable temperature profiles experienced by individuals in the field, there is good evidence to suggest laboratory‐based indices of cold tolerance to static temperatures can be surrogates for field survival under more variable conditions (Bale & Hayward, 2010). Larvae were then transferred to a 5 °C controlled cabinet for 2 days, and larval survival determined by movement in response to mechanical stimulus. Live larvae were transferred to *P. pratensis* host plants (larvae from one treatment group on each plant, maximum *n* = 10 larvae) and survival rates to pupation and eclosion recorded. Development time was calculated as the number of days from removal from experimental treatment to pupation. A ‘control’ group of 10 larvae did not experience subzero temperatures but were kept at 5 °C for 8 days, the duration of the longest cold exposure treatment, and performance variables compared with experimental larvae. Data for larval survival, development time, pupal mass and growth rates (based on mean values per host plant pot) were analysed using analysis of covariance (ancova), with temperature as a fixed factor and duration of exposure as a covariate. Survival was calculated relative to survival of the control group as proportion of larvae alive in treatment group/proportion alive in control group at each developmental stage.

## Results

### Spatial variation in habitat associations

Estimates of *W*_*i*_ varied between 0.56 and 0.81 showing that the study species is more strongly associated with closed (woodland) habitats than open habitats (*W*_*i*_ > 0.5) throughout its British range, but varies in the strength of this association (Fig. 1c). Separate logistic regressions for each of the four climate variables on the strength of *P. aegeria*'s association with woodland showed that the species is more strongly associated with woodland in places with cooler winters and summers (Table 1). When all four climate variables and their interactions were included in the same model, the terms that remained in the minimal adequate model were summer temperature, summer rainfall and the interaction between these main effects (Table 1). The interaction reveals that in places with cooler than average (14.9 °C) summers, there is no relationship between the strength of the butterfly's association with woodland and summer rainfall (logistic regression: slope coefficient = 0.002, *t*_1,13_ = 1.44, *P *=* *0.173). In places with warmer than average summers, however, the butterfly is more strongly associated with woodland in dry places than wet places (logistic regression: slope coefficient = −0.002, *t*_1,17_ = −2.16, *P *=* *0.045). This model predicts observed variation in the strength of association with woodland well (*R* ^2^ observed vs. fitted values = 0.74; Fig. 1c, d).

**Table 1:** Relationships between climate and spatial variation in *Pararge aegeria*'s associations with woodland

Independent variable(s) in model	df	Intercept	Slope	Slope SE	*t*‐value	*P*‐value	Moran's *I*
Summer temperature	32	1.633	−0.080	0.029	−2.770	0.009	Sig
Winter temperature	32	0.795	−0.089	0.032	−2.776	0.009	Sig
Summer rainfall	32	0.202	0.001	0.0007	1.582	0.123	Sig
Winter rainfall	32	0.345	0.0002	0.0003	0.677	0.503	Sig
Minimal adequate model	30	−1.572					Nonsig
Summer temperature			0.156	0.098	1.599	0.120	
Summer rainfall			0.021	0.007	3.076	0.004	
Interaction term			−0.002	0.0005	−3.320	0.002	

### Temporal variation in habitat associations

**Figure 2:**
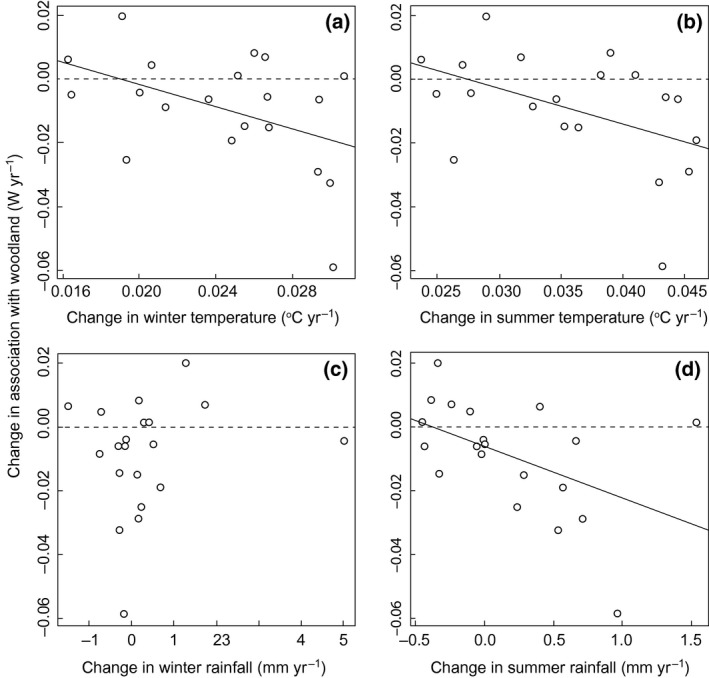
Relationship between change in *Pararge aegeria*'s association with woodland (*W*_*ij*_) and change in climate over time. Climate variables are change between 1970 and 2006 in (a) mean winter temperature [change in *W*_*ij*_ = 0.033–1.729 (change in winter temperature), *P = *0.039, *R* ^2^ = 0.20], (b) mean summer temperature [change in *W*_*ij*_ = 0.030–1.108 (change in summer temperature), *P = *0.048, *R* ^2^ = 0.22], (c) total winter rainfall and (d) total summer rainfall [change in *W*_*ij*_ = −0.006 to 0.016 (change in summer rainfall), *P = *0.030, *R* ^2^ = 0.24]. Each point represents the habitat and climate trend for one 100 km × 100 km grid square. The butterfly's association with woodland has weakened most rapidly in places where winter and summer temperatures and summer rainfall have increased most.

The association of *P. aegeria* with its favoured woodland habitat has declined in more 100 km grid squares than it has increased (decrease = 13 squares, increase = 7 squares), and the association has declined most rapidly in 100 km grid squares where average winter temperature has increased most (Fig. 2a), where summer temperature has increased most (Fig. 2b), and where summer rainfall has increased most (Fig. 2d; Table 2). Thus, the relationships between climate and habitat associations are similar in both space and time, driven primarily by summer and winter temperature and summer rainfall.

**Table 2:** Relationships between change in *Pararge aegeria*'s associations with woodland over time and change in climatic conditions over time

Independent variables(s) in model	df	Intercept	Slope	Slope SE	*F* ratio	*P*‐value	Moran's *I*
Change in winter temperature	18	0.033	−1.729	0.816	4.946	0.048	Nonsig
Change in summer temperature	18	0.030	−1.108	0.450	4.949	0.039	Nonsig
Change in winter rainfall	18	−0.010	0.002	0.003	0.441	0.515	Nonsig
Change in summer rainfall	18	−0.006	−0.016	0.006	5.587	0.030	Nonsig
Minimal adequate model for spatial analysis	16	0.025					Nonsig
Summer temperature			−0.898	0.597	5.486	0.032	
Summer rainfall			−0.014	0.050	3.955	0.064	
Interaction term			0.009	1.28	0.000	0.995	

### Field experiments

Over winter, the study species performed better in closed (woodland) habitat, where larval development time was significantly shorter, larval growth rates were significantly faster, and pupae were significantly heavier, compared with grassland (Table 3). There was no significant difference in overwinter larval survival between habitats (Table 3), although there was high variance among pots (range 0–86% surviving). Temperature data showed the severity of cold exposure was reduced in woodland, compared with grassland, as were the number of freeze–thaw cycles and magnitude of fluctuations in temperature, although the absolute duration of exposure to freezing temperatures was similar between habitats (Table S1).

**Table 3:** Summary of differences in larval performance in woodland and grassland in winter and summer field experiments and results of statistical tests for differences between habitats

Season	Insect performance variable	Mean woodland (standard error)	Mean grassland (standard error)	*t*‐value	df	*P*‐value
Winter	Survival (percentage)	36.6 (5.03)	30.8 (2.82)	−0.866	45	0.391
	Development time (weeks)	30.8 (0.27)	31.8 (0.19)	2.880	43	0.006
	Pupal mass (mg)	181.6 (5.89)	153.8 (3.74)	3.919	42	<0.001
	Growth rate (mg week^−1^)	5.88 (0.21)	4.85 (0.13)	−4.262	42	<0.001
Summer	Survival (percentage)	52.5 (0.07)	47.5 (0.07)	−0.503	39	0.618
	Development time (days)	23.0 (1.34)	27.4 (1.26)	−2.114	14	0.053
	Pupal mass (mg)	142.6 (5.25)	126.8 (4.95)	1.917	14	0.076
	Growth rate (mg day^−1^)	6.30 (0.50)	4.73 (0.27)	3.031	14	0.009

In summer, the study species also performed better in closed (woodland) habitats, where larval growth rates were significantly higher, and there was some evidence that development time was shorter and pupae were heavier, compared with grassland (Table 3). As in winter, there was no difference in larval survival rates between habitats and high variance in survival rates among pots (range, 0–100% surviving; Table 3). Water content in wild grass was significantly lower in open (grassland mean = 50.5%) than in closed habitats (woodland mean = 66.7%; *t*‐test: *t* = −10.25, df = 163 *P *<* *0.0001), although there was no significant difference in potted plants (grassland mean = 34.3% water; woodland mean = 45.9%; Mann–Whitney *U*‐test: *Z* = −1.257, *n* = 38, *P *=* *0.217). GDD5 was higher in open vs. closed habitats (Table S1).

### Laboratory experiments

**Figure 3:**
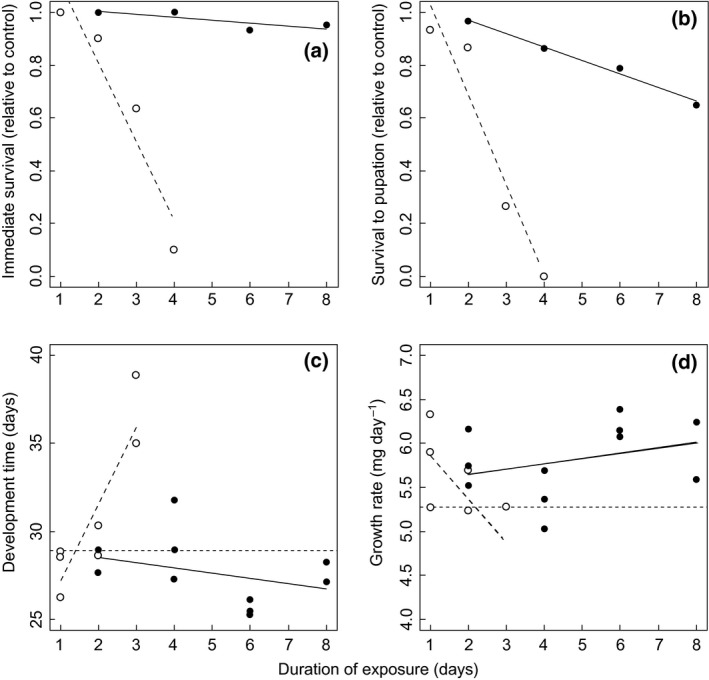
Lethal and sublethal effects of cold exposure on *Pararge aegeria* larvae in laboratory experiment. Solid lines and closed symbols are larvae exposed to −5 °C, and long dashed lines and open symbols are larvae exposed to −10 °C. (a) Survival 2 days after stress treatment was terminated; (b) survival to pupation; (c) development time to pupation (days); and (d) growth rate (mg per day) following different cold exposure durations. For (a and b), survival rate is calculated as a proportion of survival rates of the control group. For (c and d) the horizontal short dashed lines show the average values for control groups.

Immediate larval mortality increased with the duration of cold exposure and showed a significant interaction between exposure temperature and duration (ancova: exposure temperature: *F*_1,4_ = 3.855, *P *=* *0.121; duration of exposure: *F*_1,4_ = 31.105; *P *=* *0.005; interaction term: *F*_1,4_ = 26.876; *P *=* *0.007; Fig. 3a). Similar effects were found for survival to pupation (ancova: exposure temperature: *F*_1,4_ = 2.356, *P *=* *0.200; duration: *F*_1,4_ = 50.947; *P *=* *0.002; interaction: *F*_1,4_ = 27.650, *P *=* *0.006; Fig. 3b), eclosion success (ancova: exposure temperature: *F*_1,4_ = 0.923, *P *=* *0.391; duration: *F*_1,4_ = 138.185; *P *<* *0.001; interaction: *F*_1,4_ = 67.390, *P *<* *0.001), larval development time (ancova: exposure temperature: *F*_1,14_ = 7.066, *P *=* *0.019; duration of exposure: *F*_1,14_ = 18.588; *P *<* *0.001; interaction: F_1,14_ = 24.265, *P *<* *0.001; Fig. 3c) and larval growth rates (ancova: exposure temperature: *F*_1,14_ = 2.611, *P *=* *0.128; duration of exposure: *F*_1,14_ = 4.345; *P *=* *0.056; interaction: *F*_1,14_ = 7.190, *P *=* *0.018; Fig. 3d). Significant interaction terms between exposure temperature and duration in these analyses revealed that detrimental impacts of cold temperature were particularly evident in −10 °C treatments (Fig. 3). No significant results were obtained for pupal mass (ancova: exposure temperature: *F*_1,14_ = 0.075, *P *=* *0.789; duration of exposure: *F*_1,14_ = 0.624; *P *=* *0.443; interaction: *F*_1,14_ = 0.806, *P *=* *0.385).

## Discussion

Our focal study species altered its habitat associations in space and over time, in line with variation in similar aspects of the climate. These patterns in habitat associations reflect ecophysiological responses of the study species to microclimatic differences in its favoured (woodland) habitat vs. more open habitats. Taken together, the results suggest that interactions between macro‐ and microclimate can produce complex patterns of habitat associations as species respond physiologically to different aspects of the climate.

The data confirm that the study species varies in its habitat associations from being predominantly a habitat specialist (up to 81% of locations in which *P. aegeria* has been recorded in woodland, controlling for variation in recorder effort between habitats) in places with marginal climates, to being a relative habitat generalist (down to 56% association with woodland) in places with more favourable macroclimates. In this case, we showed increased generalization in places with warm winters compared with cool winters and places with warm and wet summers compared with warm and dry summers. Thus, species may not show simple relationships between habitat associations and climate throughout their ranges (i.e. increasing specialization towards range boundaries; Brown, 1984; Thomas *et al*., 1999), but may have complex patterns of habitat associations, even within the core of their range, as they respond to different components of the climate. The study species has also undergone substantial changes in the relative distribution of individuals between its favoured woodland habitat and nonwoodland habitats over time and has relaxed its habitat associations most in areas where winter and summer temperature and summer rainfall have increased most. These results suggest that multiple climate factors can determine changes in species' habitat associations over time and that temporal changes may differ throughout a species' range, including at its range core.

These results were derived from a single land cover map, but woodland cover in Britain has changed over the period of the study (1970–2009; Forestry Commission, 2003) which could potentially affect our results. However, woodland cover, on average, increased during this time and so we would expect an opposite trend (an increase in the proportion of individuals recorded in woodland because more woodland is available) if land cover change was driving our results. Thus, we conclude that our findings are primarily due to the effects of climate variation, rather than any changes to woodland availability.

Field and laboratory experiments support the hypothesis that spatial and temporal variation in habitat associations can be mediated by the effect of habitat structure on the microclimate that an individual organism experiences. Larval performance was consistently higher in closed than open habitats, with woodland larvae growing faster and achieving higher pupal mass in a shorter development period than grassland larvae (Table 3). Over winter, temperatures recorded in open habitats were colder than in closed habitats (−8.9 °C in grassland and −5.1 °C in woodland, comparable to the differences in minimum temperatures between habitats found in previous studies; e.g. Suggitt *et al*., 2011). It is possible that colder temperatures in open habitats induce longer and more intense diapause (Coleman *et al*., 2014). Alternatively, slower development in open habitats may have been a result of greater chill injury (Turnock *et al*., 1985). This conclusion was supported by our laboratory experiments where larval development time increased with severity and duration of cold exposure. Grassland larvae may also have been harmed by exposure to greater diurnal fluctuations in temperature (Table S1) due to more rapid cooling and hence lower cold tolerance of individuals (Kelty & Lee, 1999; Woodman, 2010), or increased physiological costs associated with rapid cold hardening processes (Overgaard *et al*., 2007). Warmer daytime temperatures in grassland could also be harmful because individuals resume feeding above the 6 °C development threshold of *P. aegeria* (Blakeley, 1996) and so are more likely to have damaging ice nucleators (plant fragments) in their gut (Woodman, 2010) when temperatures drop below freezing at night. However, return to warmer daytime temperatures could also help individuals recover from chill injury (Rinehart *et al*., 2011). Over summer, the water content of wild grass samples was significantly lower in open habitats than in woodland, supporting the idea that host plant desiccation is higher in open habitats. Thermal availability for development was greater in open habitats and so the most plausible explanation for poorer larval performance in open grassland habitat is that it is impaired by desiccated host plants, given that the water content of plant material is a strong predictor of the growth rates of chewing insects (Scriber & Slansky, 1981). In summary, both winter and summer performances were better in the species' favoured (woodland) habitat, but for different microclimatic reasons in the two seasons.

Increased larval growth rates, reduced development time and greater pupal weights in the species' favoured closed (woodland) habitat are likely to lead to higher species' population growth rates. For example, higher pupal mass in woodland (1.12 times in summer, 1.18 in winter) is likely to lead to increased adult fecundity (Karlsson & Wickman, 1990) and hence higher maximum potential population growth rates. Faster development times in woodland (by 1 week in winter and by 4.4 days in summer) could result in increased population growth rates due to reduced risk of mortality prior to adulthood (Pollard, 1979), or completion of additional generations or life stages due to the butterfly's flexible life history (Shreeve, 1986). In combination, these factors are likely to affect relative population growth rates in woodland vs. grassland but do not represent absolute barriers to survival outside woodland. Rather, quantitative reductions in performance may be sufficient to decrease the proportion of *P. aegeria* found outside its most favoured habitat in climatically marginal regions, consistent with observations from distribution data that *P. aegeria* is increasingly but not completely restricted to woodland in the coolest and hottest and driest parts of its range. Similarly, the butterfly's association with woodland has weakened most over time where winter temperatures and summer rainfall have increased. Changes in climatic conditions in these regions are likely to have reduced the sublethal effects of cold and host plant desiccation in open habitats, resulting in relaxation of habitat associations and *P. aegeria* occupying open habitats. Thus, population growth rates can differ between habitats due to the effect of microclimate on individual fitness, and this can interact with macroclimate to drive spatial and temporal variation in a species' habitat associations.

Larvae used in field and laboratory experiments were all offspring of individuals captured in woodlands. There is some evidence to suggest that individuals from agricultural landscapes in continental Europe show behavioural and physiological differences to those from woodland landscapes, suggesting some adaptation to the different microclimatic conditions and resource availability in these habitats (Berwaerts *et al*., 1998; Karlsson & Van Dyck, 2005; Merckx & Van Dyck, 2006; Merckx *et al*., 2008). It is possible, therefore, that responses in the physiological variables measured here could be different in individuals captured from open habitats, and this deserves more study.

## Implications and conclusions

Previously, species' habitat associations have been shown to become narrower towards range margins, resulting in smaller, more isolated populations as a smaller fraction of the landscape is available to the species (Thomas *et al*., 1999). Here, we show that patterns of habitat associations across a species' range can be complex as they respond to different aspects of the climate. Knowledge of spatial variation in habitat associations is important because, in patchy landscapes, this determines patch size and connectivity and hence the viability of populations. Thus, species may be more vulnerable in some parts of their range than others, and conservation management may need to be adapted to a species' specific requirements in different regions. Similarly, it is important to consider that habitat associations may change over time throughout a species' range in response to climate change, and not only at the margins. As conditions become more favourable in some regions, more of the landscape can be occupied and populations could become larger and less fragmented. At species' leading range margins, greater habitat availability can increase rates of range expansion as population sizes increase and colonization distances to new habitat decrease (Wilson *et al*., 2010). Habitat availability and populations will decline in other regions, however, as conditions become less favourable, for example, due to increasing drought conditions.

Maintaining fine‐scale heterogeneity within habitat types has previously been highlighted as a conservation method to help species respond and adapt to climate change by ensuring a range of microclimates are available within a given habitat type, and hence increasing the probability of a population persisting under a range of climatic conditions. Here, we have shown that, for some species with sufficient flexibility in their life history, variation in their use of broad habitat types over time is also possible. Hence, maintaining or (re)creating connectivity between broad habitat types (e.g. open and closed habitats), as well as between more specialist habitats (e.g. grass swards of different heights), may be critical for a species to persist in a particular landscape (Oliver *et al*., 2010).

## Supporting information


**Table S1.** Microclimate data from field experiments.

**Table S2.** Comparison of severity and duration of larval cold exposure in field and laboratory experiments.

**Methods S1.** Additional information relating to the design of field and laboratory experiments.
