# Supplementary material for: Macro‐ and microclimatic interactions can drive variation in species' habitat associations
Source: Glob Chang Biol. 2015 Nov 26;22(2):556–66. doi: 10.1111/gcb.13056 (PMC4991288; doi:10.1111/gcb.13056)
Supplement: Supplementary file 1 — Table S1. Microclimate data from field experiments. [file GCB-22-556-s001.docx]

**Table S1** Microclimate data from data loggers placed 30 cm above the ground in woodland and grassland sites which collected data every hour from 23^rd^ September 2008 to 16^th^ June 2009 for winter experiment and 6^th^ August 2009 to 30^th^ September 2009 for summer experiment. Values are the average of all loggers in woodland and grassland.

| **Climate variable** | **Woodland** | **Grassland** |
| --- | --- | --- |
| **Winter experiment** |  |  |
| Absolute minimum temperature (°C) | -5.1 | -8.9 |
| Mean daily minimum temperature (°C) | -0.2 | -4.9 |
| Hours spent below 0 °C | 444 | 453 |
| Freezing day degrees below 0 °C | -614 | -911 |
| Number of freeze-thaw cycles | 62.5 | 86 |
| Mean diurnal range (°C) | 7.9 | 13.9 |
| Growing degree days above 5 °C | 803 | 963 |
| **Summer experiment** |  |  |
| Growing degree days above 5 °C | 506 | 621 |
