# Supplementary material for: Macro‐ and microclimatic interactions can drive variation in species' habitat associations
Source: Glob Chang Biol. 2015 Nov 26;22(2):556–66. doi: 10.1111/gcb.13056 (PMC4991288; doi:10.1111/gcb.13056)
Supplement: Supplementary file 2 — Table S2. Comparison of severity and duration of larval cold exposure in field and laboratory experiments. [file GCB-22-556-s002.docx]

**Table S2** Duration of below 0 °C larval exposure and accumulated degrees below 0 °C in experimental treatments in laboratory experiment.

| **Duration of exposure (days)** | **1** | **2** | **3** | **4** | **5** | **6** | **7** | **8** |
| --- | --- | --- | --- | --- | --- | --- | --- | --- |
| **Hours spent below**  **0 °C** | 24 | 48 | 72 | 96 | 120 | 144 | 168 | 192 |
| **Freezing degrees days below**  **0 °C at -5 °C** | - | 240 | - | 480 | - | 720 | - | 960 |
| **Freezing degree days below**  **0 °C at -10 °C** | 240 | 480 | 720 | 960 | - | - | - | - |
