# Supplementary material for: Macro‐ and microclimatic interactions can drive variation in species' habitat associations
Source: Glob Chang Biol. 2015 Nov 26;22(2):556–66. doi: 10.1111/gcb.13056 (PMC4991288; doi:10.1111/gcb.13056)
Supplement: Supplementary file 3 — Methods S1. Additional information relating to the design of field and laboratory experiments. [file GCB-22-556-s003.docx]

**SUPPORTING INFORMATION**

***Pararge aegeria* distribution and life history**

The speckled wood butterfly (*Pararge aegeria*) is widely distributed throughout Europe, North Africa and east to the Urals (Asher *et al*., 2001). It has undergone distributional shifts over the past 200 years in Britain (Asher *et al*., 2001) and elsewhere at its northern range margin (Parmesan *et al.*, 1999). For most of the nineteenth century it was widely distributed throughout Britain, reaching as far north as central Scotland, but at the end of the nineteenth century and beginning of the twentieth century its range contracted and it became restricted to south-west England and Wales, as well as to a localised area of western Scotland (Downes, 1948; Asher *et al*., 2001; Hill *et al*., 2001). In the 1920s the butterfly’s range began to re-expand (Asher *et al*., 2001) and this has continued in the 21st century, although it has not yet recolonised all of its previous distribution (Fox *et al*., 2006).

Voltinism of *P. aegeria* varies throughout its range in relation to temperature, with continuous generations in northern Africa and southern Spain through to one generation per year in central Sweden (Nylin *et al*., 1995). It is unusual amongst butterflies in that in Britain and southern Sweden it can overwinter in both larval and pupal life stages. Overwintering larvae do not enter a true diapause in Britain, but individuals can resume feeding and continue to develop when temperatures rise above their development threshold of around 6°C (Blakeley, 1996), unlike those in southern Sweden where conditions are colder and larvae enter a “true” diapause (Wiklund and Friberg, 2011). Variation in over wintering development stages of individuals and the ability of larvae to resume feeding when conditions are suitable over winter, results in a protracted emergence of adults in spring and through into early summer. The earliest spring-emerging adults (which over-wintered as pupae) lay eggs which commonly develop through to generate another adult generation in late summer or early autumn; these individuals will then lay eggs and give rise to over-wintering larvae. In contrast, the early summer adults (which over-wintered as larvae) give rise to progeny that will commonly overwinter as pupae. The species shows both temporal and geographic variation in patterns of emergence (Hodgson *et al*., 2011), with a third adult emergence in autumn more likely in warm years (Shreeve, 1986) and more common in the south of the UK (Asher *et al.*, 2001). Developmental pathway (direct development, larval diapause or pupal diapause) is determined by photoperiod and temperature cues (Shreeve, 1986; Nylin *et al*., 1989, 1995; Wiklund and Friberg, 2011).


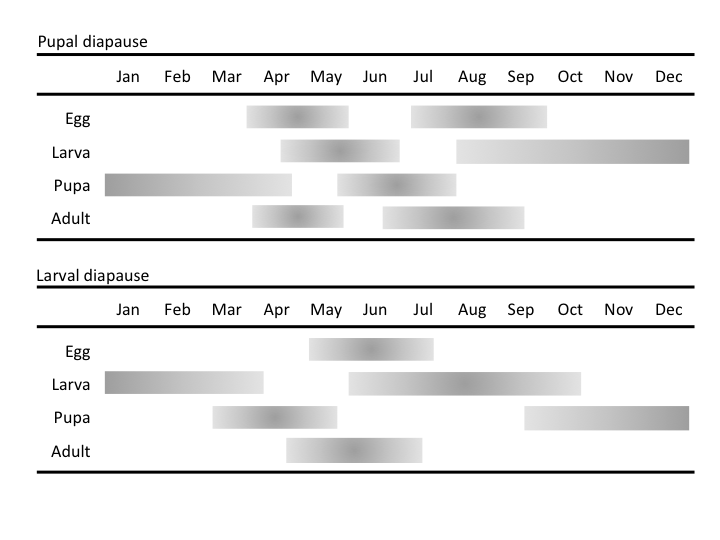


**Fig. S1.** Life cycle of *P. aegeria* in Britain. Typical developmental pathways of individuals that overwinter as larvae or pupae, and their offspring, are displayed separately. However, differences in development times due to microclimatic variation mean that some individuals may follow different pathways. For example, the fastest developing individuals that overwintered as larvae may produce rapidly-developing individuals that are able to complete a full summer generation with adults that emerge in late summer.

*P. aegeria. ssp. tircis* occurs throughout the butterfly’s British range with the exception of northern and western Scotland, where *P. aegeria ssp. oblita* is present, and on the Isles of Scilly where *P. aegeria ssp. insula* occurs. All three races can overwinter as either larvae or pupae.

Females lay eggs singly on a range of grass species including cocksfoot (*Dactylis glomerata*), false brome (*Brachypodium sylvaticum*), Yorkshire fog (*Holcus lanatus*) and couch grass (*Elytrigia repens*) (Asher *et al*., 2001). These grasses are all common and widespread species throughout Britain and found in a range of habitat types, including grassland and woodland (Stace, 1991). It is unknown whether different hosts are preferred in different parts of the butterfly’s range.

It is unknown to what extent *P. aegeria* adults move between habitat patches. Hill *et al*. (2003) estimated the maximum dispersal rate of *P. aegeria* adults to be 1.03-2.7 km per year from rates of climate-related range shifts. However, the average dispersal distance of individuals within the range is likely to be reduced compared with the invasion front where individuals have evolved increased dispersal abilities, and where expansion rates may be determined by the movements of the most vagile individuals (Hill *et al*., 1999; Hughes *et al*., 2003). Data from mark-release-recapture of individuals in Belgium showed that many individuals fly less than 300 m and only a few more than 1000 m (Berwaerts *et al*., 1998). In the UK, it is unknown whether there are open habitat and woodland populations or individuals move between the two habitats. Studies from Europe suggest some physiological differences between individuals from woodland and agricultural landscapes (Berwaerts *et al*., 1998; Karlsson & Van Dyck, 2005; Merckx & Van Dyck, 2006; Merckx *et al*., 2008). However, individuals from both landscapes orientate towards woodland suggesting they will use this habitat type if available (Merckx & Van Dyck, 2007; Ockinger & Van Dyck, 2012).

**Field experiments**

*Experimental design*

Adult female *P. aegeria* were collected from Bishop Wood, near Selby, North Yorkshire (UK Ordnance Survey Grid Reference SE5533) in August 2008 and July 2009. Wild-collected adults were kept separately on pots of *Poa pratensis* plants for egg-laying in greenhouses at the University of York. Grass seed was sourced from Emorsgate Seeds (Kings Lynn, UK) and grown in Levington F2 compost with sand. Pots were watered daily. Second instar larvae were transferred onto new plants grown in the same conditions and then pots with larvae were transferred to field sites. Pots were covered with netting to prevent grazing of grass plants, predation and parasitism of larvae, and larval movement off the experimental pots.

(1) Winter experiment

Pots with larvae were placed at a woodland site (Bishop Wood, OS grid ref SE5533), and a grassland site (Wistow, OS grid ref SE6035). At each site, pots were placed 1.5 m apart along 2 transects (20 pots per transect), and dug in so that the plant pot was flush with the surface of the soil. There was a split-brood design, and larvae from each female (n = 40 females) were split evenly between pots placed in woodland and grassland. Pots contained 5 to 15 larvae, depending on the number of eggs laid by females.

(2) Summer experiment

Pots with larvae were set up in three woodlands (Bishop Wood, OS grid ref SE5533; the Grange, OS grid ref SE6957; Skipwith Common, OS grid ref SE6637) and three grassland sites (Wistow, OS grid ref SE6035; University of York campus, OS grid ref SE6150; Skipwith Common). Pots (seven per site) were placed 1.5 m apart along one transect per site (n = 42 pots in total). Insufficient females laid enough eggs for a split brood design and so eggs from each female (n = 19 females) were split evenly between treatments but assigned randomly to pots within treatments, with all pots receiving 5 larvae.

*Microclimate measurements*

Temperature data loggers were placed within the netting surrounding the pot and took hourly temperature readings. Loggers were wrapped in foil to minimise the effect of direct solar insolation. These were iButton data loggers (model DS1922L), accurate to 0.4 °C within the temperature ranges experienced in the experiment.

Over winter, severity of cold exposure was measured as (1) the absolute minimum temperature reached in each habitat and (2) mean daily minimum temperature in each habitat. Duration of cold exposure was measured as (3) hours spent below 0 °C. (4) Freezing degree days (FDD0 calculated by summing all hourly temperature readings below 0 °C) was calculated as a measure of the combination of severity and duration of exposure. Temperature fluctuations were measured as (5) the number of freeze-thaw transitions (the number of times temperature went from above to below zero) and (6) mean diurnal temperature range (daily maximum temperature – daily minimum temperature). Over winter and summer, growing degree days above 5 °C (GDD5) was calculated as a measure of thermal availability for development; a higher GDD5 should result in faster rate and shorter development time.

Due to failure or loss of many of the data loggers over winter, data from only 2 loggers in woodland and 4 in grassland were available, and summary data (average values from successful loggers) are presented in Table S1.

*Measures of insect performance*

Upon pupation, individuals were collected and fresh mass measured on the same day using a Sartorius balance accurate to 0.1 mg. For individuals from the winter experiment, we also measured adult dry mass. Adult butterflies were frozen within 24 hours of eclosion, then dried in an oven at 60 °C for 24 hours, and weighed on a Sartorius balance accurate to 0.1 mg. Adult dry mass was strongly correlated with pupal mass (n = 41, R^2^ = 0.73) and is known to be positively correlated with fecundity in *P. aegeria* (Karlsson & Wickman, 1990; but see Hughes *et al.*, 2003). Therefore, pupal mass was used as a measure of potential fecundity.

*Host plant water content measurements*

A sample of grass measuring 3 cm in diameter in total was cut, flush with the soil surface, from each of the potted plants at the end of the experiment. Samples of grass were also taken from the vicinity of the experiment in order to determine whether patterns seen in the potted plants were replicated in the wild. Samples were taken from grass growing closest to the point 2 m and 4 m perpendicular to each pot on both sides of the transect. Samples of grass measuring 3 cm in diameter in total were taken and were cut flush with the soil surface. Samples were weighed, dried in an oven at 60 °C for 24 hours, and then weighed again to compute water content.

**Laboratory experiment**

*Experimental design*

We investigated the lethal and sublethal effects of severity and duration of cold exposure on *P. aegeria* larvae in the lab. Larvae which did not pupate before the end of the summer field experiment were left on *P. pratensis* plants outdoors in York over winter. In March 2010, individuals were transferred to controlled cabinets at 26 °C and 16L:8D photoperiod to pupate and for adults to emerge. 15 pairs of adult female and male butterflies were then placed in cages in the lab. at 26 °C and 16L:8D to mate. Males were removed after 24 hours and females left in netting cages with *P. pratensis* plants to lay eggs. Eggs were transferred to controlled cabinets at 13 °C and 12L:12D photoperiod on *P. pratensis* plants and larvae were left to develop until the end of May 2011 when individuals had reached the third instar developmental stage (determined by measuring the head capsule; Blakeley, 1996). Plants were watered regularly, and replaced as required to ensure larvae had ample fresh leaves to feed on. *P. aegeria* has three potential developmental pathways: direct development, pupal overwintering and third instar larval overwintering. The photoperiod and temperature conditions chosen have previously been shown to induce larval overwintering in *P. aegeria* from Britain (Nylin *et al.*, 1989), and hence the larvae used for the cold exposure experiments were of the type that would normally be exposed to winter cold. Larvae from the same female were split evenly between treatments.

Upon removal from cold exposure, mortality was not assessed immediately because individuals could be in chill coma. Live larvae were transferred to *P. pratensis* for 18 days at 15 °C, and then transferred to 20 °C. Newly pupated individuals were weighed 24 hours after pupation. Pupae were then kept in pots with moistened filter paper to prevent desiccation.

**References**

Asher JM, Warren M, Fox R, Harding P, Jeffcoate G, Jeffcoate S (2001) *The millennium atlas of butterflies in Britain and Ireland*. Oxford University Press, Oxford, UK.

Berwaerts K, Van Dyck H, Van Dongen S, Matthysen E (1998) Morphological and genetic variation in the speckled wood butterfly (*Pararge aegeria* L.) among differently fragmented landscapes. Netherlands Journal of Zoology, **48**, 241-253.

Blakeley D (1996) *The overwintering biology of the speckled wood butterfly* Pararge aegeria (*L.*) MPhil. University of Leeds, UK.

Downes JA (1948) The history of the speckled wood butterfly (*Pararge aegeria*) in Scotland, with a discussion of the recent changes of range of other British butterflies. Journal of Animal Ecology, **17**, 131-138.

Fox R, Asher JM, Brereton T, Roy DB, Warren M (2006) *The state of butterflies in Britain and Ireland*. Pisces Publications, Oxford, UK.

Hill JK, Thomas CD, Blakeley DS (1999) Evolution of flight morphology in a butterfly that has recently expanded its geographic range. Oecologia, **121**, 165-170.

Hill JK, Collingham YC, Thomas CD, Blakeley DS, Fox R, Moss D, Huntley B (2001) Impacts of landscape structure on butterfly range expansion. Ecology Letters, **4**, 313-321.

Hill JK, Thomas CD, Huntley B (2003) Modeling present and potential future ranges of European butterflies using climate response surfaces. In: *Butterflies: ecology and evolution taking flight* (eds Boggs CL, Watt WB, Ehrlich PR,), pp. 149-167, Chicago Press, Chicago, Illinois, USA.

Hodgson JA, Thomas CD, Oliver TH, Anderson BJ, Brereton TM, Crone EE (2011) Predicting insect phenology across space and time. Global Change Biology, **17**, 1289-1300.

Hughes CL, Hill JK, Dytham C (2003) Evolutionary trade-offs between reproduction and dispersal in populations at expanding range boundaries. Proceedings of the Royal Society of London Series B-Biological Sciences, **270**, S147-S150.

Karlsson B, Wickman PO (1990) Increase in reproductive effort as explained by body size and resource-allocation in the speckled wood butterfly, *Pararge aegeria* (L). Functional Ecology, **4**, 609-617.

Karlsson B, Van Dyck H (2005) Does habitat fragmentation affect temperature-related life-history traits? A laboratory test with a woodland butterfly. Proceedings of the Royal Society B-Biological Sciences, **272**, 1257-1263.

Merckx T, Van Dyck H (2006) Landscape structure and phenotypic plasticity in flight morphology in the butterfly *Pararge aegeria*. Oikos, **113**, 226-232.

Merckx T, Van Dyck H (2007) Habitat fragmentation affects habitat-finding ability of the speckled wood butterfly, *Pararge aegeria* L. Animal Behaviour, **74**, 1029-1037.

Merckx T, Van Dongen S, Matthysen E, Van Dyck H (2008) Thermal flight budget of a woodland butterfly in woodland versus agricultural landscapes: An experimental assessment. Basic and Applied Ecology, **9**, 433-442.

Nylin S, Wickman PO, Wiklund C (1989) Seasonal plasticity in growth and development of the speckled wood butterfly, *Pararge-aegeria* (Satyrinae). Biological Journal of the Linnean Society, **38**, 155-171.

Nylin S, Wickman PO, Wiklund C (1995) Life-cycle regulation and life-history plasticity in the speckled wood butterfly - are reaction norms predictable. Biological Journal of the Linnean Society, **55**, 143-157.

Ockinger E, Van Dyck H (2012) Landscape structure shapes habitat finding ability in a butterfly. PLoS ONE, **7**, e41517.

Parmesan C, Ryrholm N, Stefanescu C, *et al*. (1999) Poleward shifts in geographical ranges of butterfly species associated with regional warming. Nature, **399**, 579-583.

Shreeve TG (1986) The effect of weather on the life-cycle of the speckled wood butterfly *Pararge aegeria*. Ecological Entomology, **11**, 325-332.

Stace CA (1991) *New Flora of the British Isles*. Cambridge University Press, Cambridge, UK.

Wiklund C, Friberg M (2011) Seasonal development and variation in abundance among four annual flight periods in a butterfly: a 20-year study of the speckled wood (*Pararge aegeria*). Biological Journal of the Linnean Society, **102**, 635-649.
